# Body fat composition as predictive factor for treatment response in patients with newly diagnosed multiple myeloma – subgroup analysis of the prospective GMMG MM5 trial

**DOI:** 10.18632/oncotarget.19536

**Published:** 2017-07-25

**Authors:** Jonathan P. GroΔ, Johanna Nattenmüller, Stefan Hemmer, Diana Tichy, Julia Krzykalla, Hartmut Goldschmidt, Uta Bertsch, Stefan Delorme, Hans-Ulrich Kauczor, Jens Hillengass, Maximilian Merz

**Affiliations:** ^1^ University of Heidelberg, Department of Internal Medicine V, Heidelberg, Germany; ^2^ University of Heidelberg, Department of Diagnostic and Interventional Radiology, Heidelberg, Germany; ^3^ University of Heidelberg, Department of Orthopedics and Trauma Surgery, Heidelberg, Germany; ^4^ German Cancer Research Centre, Department of Biostatistics, Heidelberg, Germany; ^5^ German Cancer Research Centre, Department of Radiology, Heidelberg, Germany

**Keywords:** multiple myeloma, obesity, CT imaging, body fat composition

## Abstract

**Introduction/Background:**

Obesity is a well-known risk factor for malignant tumors and increased body mass index (BMI) is correlated to the risk of developing multiple myeloma (MM). The correlation of body fat composition with disease activity, adverse events and treatment response of MM patients has not been investigated yet.

**Patients and Methods:**

A subgroup of 108 patients from a single institution enrolled in the prospective GMMG-MM5 trial, who received a whole-body low-dose computed tomography (WBLDCT) before induction therapy, were included in this study. Body fat composition was measured in WBLDCT for each patient, divided in the compartments abdomen, pelvis, thigh and further categorized in subcutaneous (SAT) and visceral adipose tissue (VAT). The correlation of these parameters with disease activity (M protein, plasma cell count, LDH, CRAB-criteria), adverse cytogenetics, adverse events and treatment response were evaluated.

**Results:**

Significant reciprocal correlation was found between adverse cytogenetics and VAT of the abdomen and pelvis, respectively (gain 1q21: p=0.009 and p=0.021; t(4;14): p=0.038 and p=0.042). No correlation of VAT or SAT with adverse events was observed. Significant reciprocal correlation was observed between abdominal (p=0.03) and pelvic (p=0.035) VAT and treatment response. Abdominal VAT remains significant (p=0.034) independently of revised ISS stage and treatment. The BMI did not show a significant correlation with treatment response or investigated cytogenetics.

**Conclusion:**

Based on the clinically relevant difference in treatment outcome depending on VAT and SAT, excessive body fat of abdomen and pelvis might be a predictive factor for poor treatment response. Further influences in this context should be considered as well, e.g. chemotherapy dosing and body fat metabolism. Further studies are necessary to investigate this hypothesis.

## INTRODUCTION

Multiple Myeloma (MM) is a malignant plasma cell disease which leads to the death of around 63 000 patients worldwide each year. [1] Despite major progress in MM treatment [2] it still remains an incurable disease.

Knowledge about the etiology of MM is still fragmentary. Overweight and obesity has a high prevalence in the western population with the tendency to rise. [3] Obesity and excessive body fat is a risk factor for cardiovascular disease, type 2 diabetes [4], and the occurrence of malignant tumors. [5–7] For MM, studies have shown that excessive body weight, measured mainly by the body mass index (BMI), is a risk factor for incidence and mortality. [8, 9]

Current disease-specific prognostic factors, like cytogenetic abnormalities [10] and the International Staging System (ISS) [11] play a major role in the course of MM. [12, 13] Compared to disease-specific prognostic factors, such as ISS and cytogenetics, also host-specific factors, such as male sex, age, black race and prior history of Monoclonal Gammopathy of Undetermined Significance influence outcome of MM patients. Among these host-specific risk factors, obesity and body fat composition would be a modifiable variable but have not been studied in symptomatic MM patients yet. This stresses the importance of further studies concerning the interactions of obesity and MM.

Whole-body low-dose computed tomography (WBLDCT) is a cornerstone in the initial work-up for newly diagnosed MM patients, since it has the highest sensitivity to detect osteolytic bone lesions. [14] Furthermore, it offers the opportunity to image and quantify the extent of visceral and subcutaneous adipose tissue as a side product.

In this study, we correlated for the first time body fat composition as assessed by WBLDCT with factors of disease activity, adverse events and treatment response in newly diagnosed MM patients enrolled in the prospective, multicenter German-Speaking Myeloma Multicenter Group (GMMG) MM-5 trial.

## RESULTS

### Disease activity

No correlation of parameters of body fat composition and established factors for disease activity (monoclonal protein, LDH and bone marrow plasma cells) were found.

**Figure 1:**
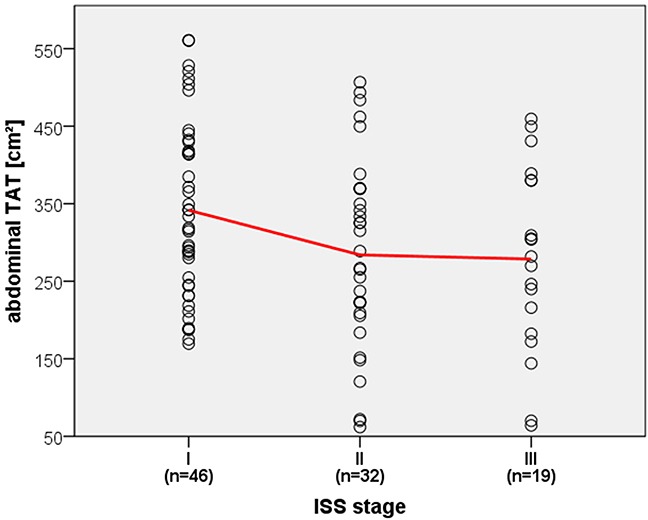
Correlation of ISS stage and abdominal TAT Dotplot displaying reciprocal correlation of ISS stages and abdominal TAT with linear interpolation line highlighted in red.

Regarding ISS stages, a reciprocal correlation was found with the abdominal TAT (*p*=0.048, mean for ISS stage I = 341.55 cm^2^ and for ISS stage III = 278.6 cm^2^) and SAT (*p*=0.036, mean for ISS stage I = 199.46 cm^2^ and for ISS stage III = 164.8 cm^2^), visualized in Figure 1.

**Figure 2:**
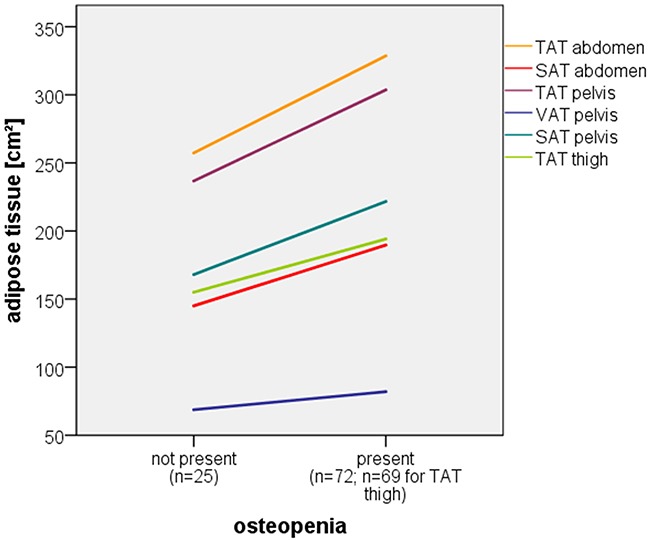
Correlation of osteopenia and multiple adipose tissue compartments Line graph displaying significant correlation of osteopenia and multiple adipose tissue compartments (linear interpolation lines of mean adipose tissue values).

No association could be found between the occurrence of CRAB criteria and body composition. However, baseline hemoglobin levels correlated significantly with abdominal TAT (*p*=0.008, *r=*0.27) and VAT (*p*=0.001, *r=*0.33) as well as pelvic VAT (*p*=0.006, *r=*0.28). Furthermore, a significant correlation was found between the occurrence of osteopenia and abdominal TAT (*p*=0.009) and SAT (*p*=0.021) as well as pelvic TAT (*p*=0.002), VAT (*p*=0.024) and SAT (*p*=0.006) and thigh TAT (*p*=0.01), displayed further in Figure 2.

### Adverse cytogenetics

A reciprocal correlation was observed between adverse cytogenetics and VAT of the abdomen and pelvis. The explored high risk cytogenetics consist of t(4;14) (*p*=0.038 for abdomen and *p*=0.042 for pelvis) and gain 1q21 (*p*=0.009 and *p*=0.021, respectively). Additionally TAT of the abdomen was significantly decreased under presence of t(4;14)(p=0.046). VAT of the abdomen was significantly decreased under presence of Del 13q14 (*p*=0.036). Del 17p13 was identified but due to the low number of positive patients (n=6/97) it was not investigated. Table 1 summarizes analysis of body compostion and cytogenetic abnormalities.

**Table 1:** Single factor analysis of variance (ANOVA) of mean visceral adipose tissue (VAT) values of the abdomen (abd.) and pelvis (pel.) subject to the presence of baseline cytogenetic abnormalities (CA). *=significant (*p*<0.05)

		Absent CA *(n)*	Present CA *(n)*	*P* value
**t (4;14)**	Abd. VAT [cm^2^]	137.12 *(77)*	98.94 *(14)*	0.038*
	Pel. VAT [cm^2^]	80.64 *(77)*	65.34 *(14)*	0.042*
**gain 1q21**	Abd. VAT [cm^2^]	147.42 *(54)*	110.35 *(29)*	0.009*
	Pel. VAT [cm^2^]	84.33 *(54)*	70.81 *(29)*	0.021*
**del 13q14**	Abd. VAT [cm^2^]	143.31 *(53)*	114.65 *(37)*	0.036*
	Pel. VAT [cm^2^]	82.81 *(53)*	72.14 *(37)*	0.056

### Adverse events

Analysis of adipose tissue parameters and adverse events, consisting of infection (*n*=29/97) and leucopenia (*n*=31/97), showed no significant correlation. Thrombocytopenia and thromboembolism were documented but due to the low number of affected patients (*n*=7/97; *n*=3/97, respectively) they were not statistically evaluated.

### Treatment response

**Figure 3:**
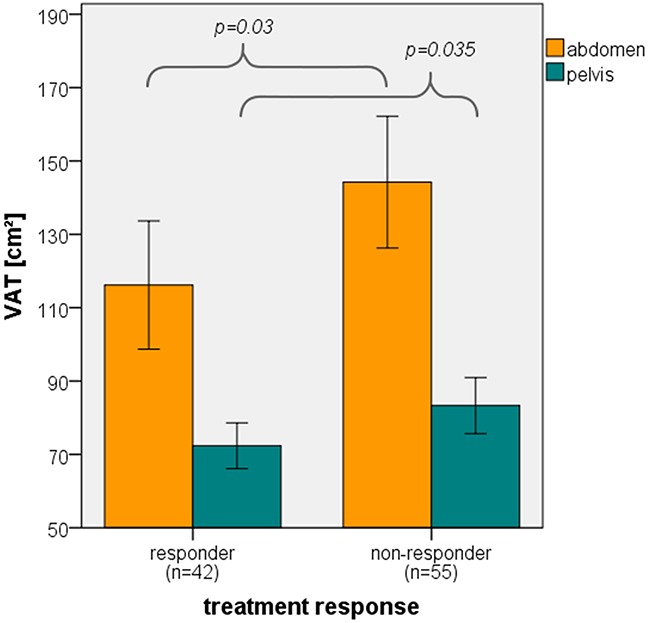
Correlation of treatment response and VAT Bar graph displaying significant correlation (ANOVA) of VAT of the abdomen and pelvis and treatment response. Error bars represent 95% confidence interval.

42 patients achieved a VGPR or better and 55 patients showed a PR or worse. Treatment response was negatively correlated to VAT of the abdomen (*p*=0.03) and pelvis (*p*=0.035). Responders thus had a lower mean VAT of the abdomen (116.17 cm^2^) and pelvis (72.36 cm^2^) than non-responders (144.22 cm^2^ and 83.3 cm^2^, respectively; Figure 3). Other adipose tissue parameters did not correlate significantly.

In the multivariate analysis, the only fat parameter remaining in the model after backward selection was the abdominal VAT. It showed a significant effect on treatment response (*p*=0.034) even if the effect was adjusted for revised ISS and treatment. Important to note is that the results of a variable selection have to be interpreted with care as the model is somewhat optimized for the underlying data set. However, in the bootstrap analysis, abdominal visceral adipose tissue was selected in more than 40% of the bootstrap replicates such that further investigations seem well-founded.

Neither the revised ISS nor the treatment showed a significant effect on the response to treatment in the multivariate model.

### Body mass index

In contrast to parameters of body fat composition, the BMI did not show a significant association with ISS (*p*=0.22), osteopenia (*p*=0.128), del13q14 (*p*=0.518), gain1q21 (*p*=0.872), t(4;14)(*p*=0.354) and treatment response (*p*=0.222). However, BMI was correlated positively with baseline hemoglobin levels (*p*=0.015).

## DISCUSSION

With the current study we show for the first time that excessive visceral adipose tissue is associated with inferior response to a bortezomib-based induction therapy in patients with newly diagnosed, symptomatic multiple myeloma. We furthermore observed a negative correlation between visceral adipose tissue and the presence of ISS stage III and high risk cytogenetics abnormalities.

Patients with high amounts of VAT of the abdomen and pelvis showed a significantly worse treatment response, statistically independent of revised ISS stage and treatment arm. Of course, these findings need to be further investigated in future studies. For example, the development of weight gain or loss over time relating to the course of disease and therapy should be evaluated. In addition, obesity is strongly associated with multiple comorbidities commonly summarized in the context of metabolic syndrome which need to be taken into account separately in order to understand more thoroughly the interaction of these diverse variables. Further influences which could be relevant in this context, chemotherapy dosing, metabolism of body fat and its interaction with drugs as well as the influence of the immune system might be considered as well. Chemotherapy dosing is mostly calculated by the body surface area and thus, obese patients receive a lower dose of chemotherapy according to their body weight than a normal weighing patient. Furthermore, it is not clear what role the metabolism of body fat especially the abdominal VAT plays in the context of pharmacodynamics and pharmacokinetics. The proteasome inhibitor bortezomib for instance decreases MM related inflammation amongst others through inhibition of NF-κB and interaction with inflammation cytokines such as tumor necrosis factor or interleukin-6. [24, 25] Other studies have shown that adipose tissue releases proinflammatory cytokines such as the above, especially the metabolically highly active visceral compartment. [26, 27] These findings could be a plausible explanation and suggest an interference of visceral adipose tissue with the mode of action of bortezomib. Since bortezomib, included in both treatment regimens in the MM5 trial, can be applied subcutaneously, these influences are not trivial. However, recent studies in relapsed and newly diagnosed MM demonstrated that higher baseline BMI does not hamper the effect of subcutaneous bortezomib. [16, 28] Since we demonstrate with our current study that VAT might have a negative impact on treatment response, further studies are warranted examining body composition rather than BMI alone.

For all analysed high risk cytogenetic abnormalities and ISS III, a reciprocal correlation was observed. Patients with high risk cytogenetic abnormalities t(4;14) and gain1q21 as well as patients with ISS III had less adipose tissue in our analysis. Weight loss is often a first clinical “B-symptom” of B-cell lymphomas and associated with poor outcome. [29, 30] Factors produced by tumor and host cells causing lipolysis include tumor necrosis factor and interleukin 6. [31] Especially, the expression of pro-inflammatory molecules like interleukin 6 has been linked to poor survival in MM, suggesting a link between more aggressive disease and loss of adipose tissue. [32]. Another cytokine associated with progression and survival in MM as well as tumor-induced cachexia is the B-cell Activating Factor (BAFF). [33] Interestingly, BAFF expression has also been associated with proliferation and disease stage according to ISS, providing a link between disease biology weight loss. [34]

**Figure 4:**
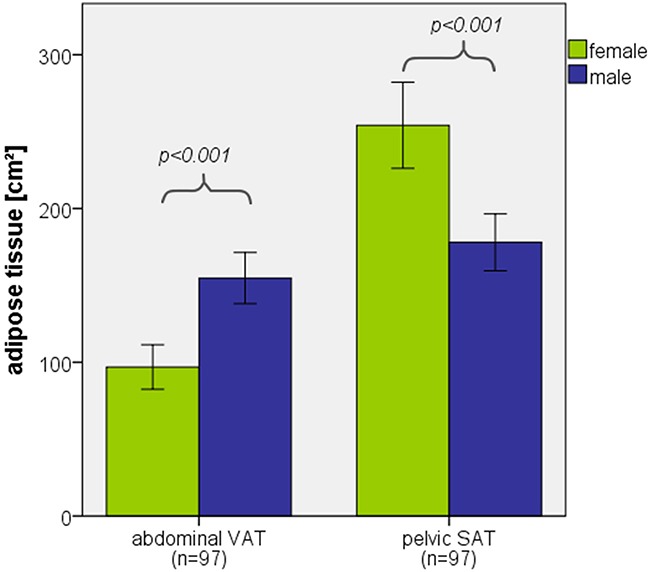
Distribution of adipose tissue among female and male sex Bar graph displaying significant difference (ANOVA) of abdominal VAT and pelvic SAT distribution among female and male sex. Error bars represent 95% confidence interval.

Besides the observed link between body compos-ition, the presence of cytogenetic abnormalities and treatment response, we observed a correlation of VAT with baseline hemoglobin levels. Studies have shown that men and women exhibit a different distribution of body fat. [35] While main body fat of women is located in the subcutaneous gluteofemoral area, men mainly accumulate body fat in the visceral abdomen, as shown for our patients in Figure 4. Men are known to have higher average hemoglobin levels. [36] Bringing those two facts together suggests, that the observed correlation between VAT and hemoglobin is merely due to a third variable, the male sex, in which both parameters are physiologically higher and thus seem to correlate.

We furthermore assume that the significant correlation between osteopenia and multiple adipose tissue parameters is rather a metabolic phenomenom than MM-related. Studies have shown that VAT, independent of MM, is a negative predictive factor for bone microarchitecture and mechanical properties in obese men. [37]

In our study, the BMI did not correlate with the treatment response and multiple other clinical and laboratory parameters, which showed a significant correlation for the WBLDCT based adipose tissue measurement. This underlines the superiority of the WBLDCT based adipose tissue measurements in comparison with the conventional measuring of weight and height for BMI calculation. Since low dose WBLDCT is a standardized staging examination for newly diagnosed MM patients, no additional examination needs to be performed for adipose tissue measurements and thus does not display additional exposure to radiation exclusively for these measurements. [38, 39] For daily clinical practice, even the measurement of one slice, preferably at the level of L3/L4, is sufficient for valid information. [40]

Furthermore, obesity is a modifiable potential predictive factor, as compared to ISS and cytogenetics. Life style changes, as a first non-invasive therapy with no major side effects and engaging in regular physical activity might have a positive effect, especially on bone health in MM patients.

In summary, we demonstrate for the first time a link between body fat composition and treatment response in patients with newly diagnosed MM. Standarized WBLDCT based adipose tissue measurement at the start of therapy might have prognostic implications and should therefore be investigated in future studies.

## MATERIALS AND METHODS

### Patients

**Figure 5:**
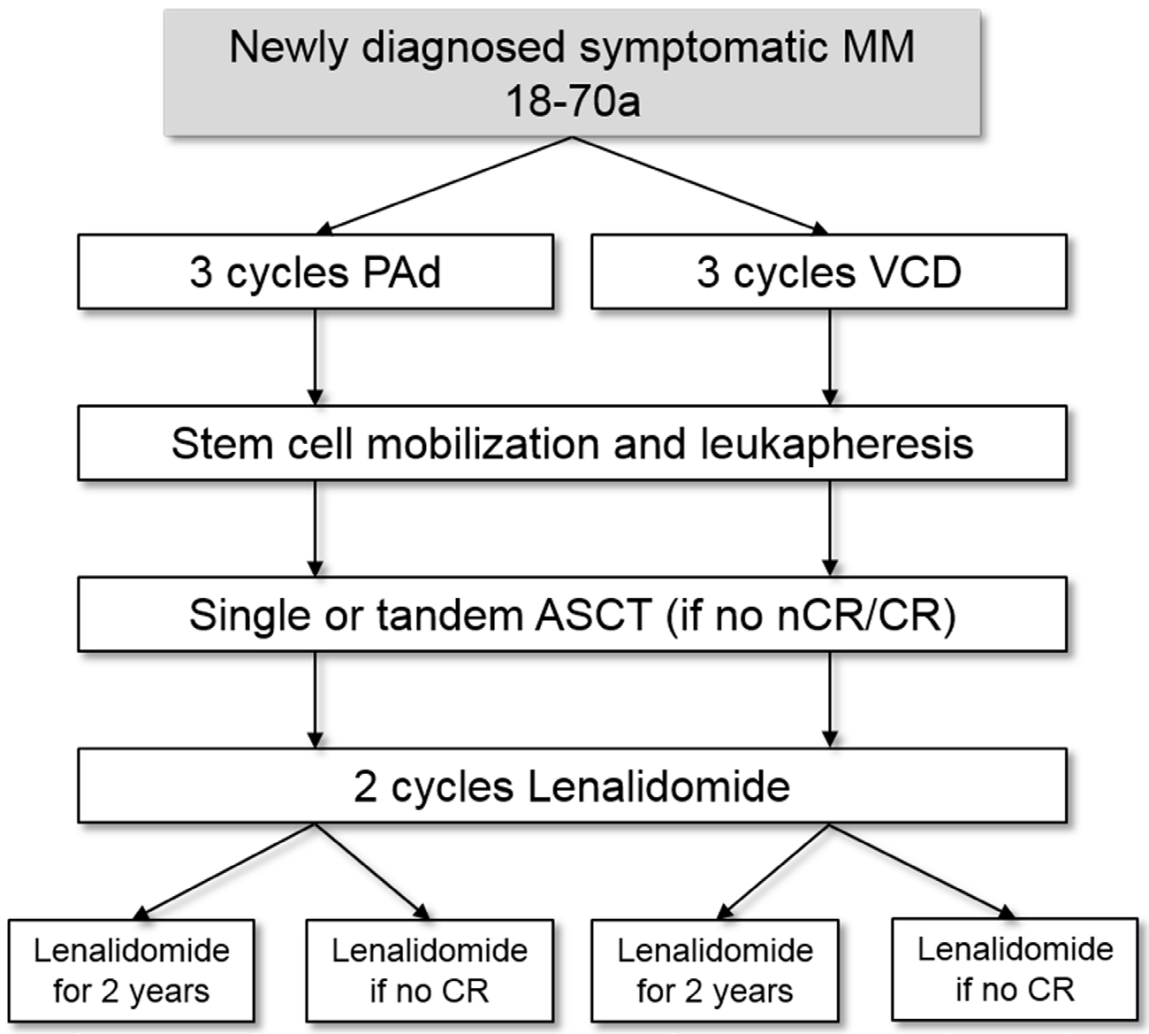
Treatment protocol of the GMMG MM5 study Flow Chart of the GMMG MM5 study. Patients were randomized to four treatment arms with either PAd (2 arms) or VCD (2 arms) induction therapy and lenalidomide maintenance therapy either for 2 years or until complete response was achieved. PAd: bortezomib-adriamycin-dexamethasone; VCD: bortezomib-cyclophosphamide-dexamethasone; ASCT: autologous stem cell transplantation; CR: complete response; nCR: near complete response.

In the GMMG MM5 trial, a prospective, randomized, open-label phase III trial (EudraCT n. 2010-019173-16), two regimens of bortezomib based induction therapy and lenalidomide consolidation followed by lenalidomide maintenance treatment are evaluated. [15] The therapy protocol is depicted more detailed in Figure 5. Patients with newly diagnosed, symptomatic, previously untreated MM were enrolled in 31 transplantation centers and 75 associated trial sites throughout Germany. The MM5 trial is being performed in accordance with the Declaration of Helsinki and the European Clinical Trial Directive (2005) and was approved by the local ethics committees of all participating institutions. [16] The trial has completed its recruitment and patients are in follow-up.

In this retrospective study, a subgroup of 108 patients enrolled at our trial site in the MM5 trial, who received WBLDCT before 3 cycles induction therapy (VCD [bortezomib, cyclophosphamide, dexamethasone] or PAd [bortezomib, adriamycin, dexamethasone]) as part of the standardized staging examination, were included. WBLDCTs of 97 patients were technically viable for the planned analysis. Exclusion criteria included incomplete image data set (n=10) or patient outside of the field of view (n=1). Data on disease activity, adverse cytogenetics, adverse events and treatment response were captured in the context of the MM5 trial. Patients´ characteristics are summarized in Table 2.

**Table 2:** Baseline characteristics of included patients

Sex	
Female *[n]*	38 (39%)
Underweight *(<18,5 kg/m^2^)*	1
Normal weight *(18,5-24,9 kg/m^2^)*	19
Overweight *(25-29,9 kg/m^2^)*	10
Obese *(≥30 kg/m^2^)*	8
Male *[n]*	59 (61%)
Underweight *(<18,5 kg/m^2^)*	0
Normal weight *(18,5-24,9 kg/m^2^)*	21
Overweight *(25-29,9 kg/m^2^)*	28
Obese *(≥30 kg/m^2^)*	10
**Demographics** (median (range))	
Age *[a]*	59 (33-70)
BMI *[kg/m^2^]*	26 (18.4-39.8)
Height *[cm]*	172 (155-197)
Weight *[kg]*	77 (49-121)
**Treatments**	
PAd *[n]*	44 (45%)
VCD *[n]*	53 (55%)
**Disease activity** (median (range))	
Monoclonal protein *[g/l]*	42 (4-102.9)
LDH *[U/l]*	192 (105-415)
Plasma cells *[%]*	40 (1-95)
ISS (I, II, III) *[n]*	46 (47%), 32 (33%), 19 (20%)
**CRAB criteria** (median (range))	
Calcium *[mmol/l]*	2.37 (1.93-3.68)
Creatinine *[mg/dl]*	0.93 (0.54-11.04)
Hemoglobin *[g/dl]*	11 (7.9-16.3)
Osteopenia (present) *[n]*	72 (74%)
Osteolysis (present) *[n]*	80 (82%)
Fracture (present) *[n]*	38 (39%)
**Adverse cytogenetics**	
T(4;14) (present) *[n]*	14/91 (15%)
Del 17p13 (present) *[n]*	5/92 (5%)
Gain 1q21 (present) *[n]*	29/83 (35%)
Del 13q14 (present) *[n]*	37/90 (41%)

### Protocol for body fat composition measurement

WBLDCTs were provided through the Picture Archiving and Communication System (PACS, Centricity® 4.1, GE Healthcare, Barrington, IL, USA) of the Department of Diagnostic and Interventional Radiology of the University of Heidelberg (DDIR, n=74) and the German Cancer Research Center Heidelberg (DKFZ, n=21), Germany. External institutions provided 4 WBLDCTs. A low dose protocol was used with a median slice thickness of 3 mm (min: 1 mm; max: 5 mm) and no contrast media was administered. The measurements were performed using the “CT volume tool” of the “SyngoVia” software (Siemens Healthcare GmbH, Munich, Germany).

In order to define adipose tissue compartments, regions of interests (ROIs) were drawn manually into images of the most cranial and caudal slice. ROIs in remaining slices were generated by the software. Each ROI was then visually inspected and adjusted manually, if necessary. An attenuation limit of −190 to −30 Hounsfield Units (HU) was assumed to represent adipose tissue. [17–19]

Measurements were divided into the three compartments: Abdomen, pelvis and thigh. Abdomen was measured from the intervertebral space between the 11^th^ and 12^th^ thoracic vertebral body (TH11 and TH12) to the intervertebral space between the 5^th^ lumbar vertebral body (L5) and the 1^st^ sacral vertebral body (S1). Pelvis was defined from the intervertebral space between L5 and S1 to the top of the symphysis. The thigh compartment was measured from the top of the symphysis to the top of the patella.

The total adipose tissue (TAT) measurements in the abdomen and pelvis were further categorized into the visceral (VAT) and subcutaneous adipose tissue (SAT), whereas in the thigh compartment only the TAT was determined.

**Figure 6:**
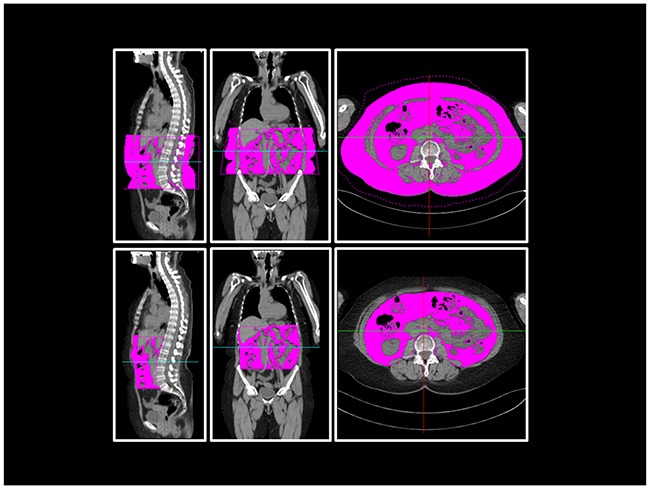
Body fat composition measurement in WBLDCT Example sagittal, coronal and axial views at the level of L3 of WBLDCT of a patient. Purple-coloured areas represent the measured total *(above)* and visceral *(below)* adipose tissue of the abdominal compartment.

The visceral compartment is circumscribed by abdominal wall muscles. In the dorsal direction, the compartment was restricted by bony structures of the spine and, further caudal, by the pelvic structure. For the pelvic measurements, the intrapelvic part of the piriformis muscle and the psoas muscle cranial of its crossing with the inguinal ligament were included. Figure 6 illustrates these measurements by means of sample CT images.

For further calculations, the measured adipose tissue volume was divided by the individual height of the equivalent compartment in order to be comparable among each other. Thus, the calculations were performed with a virtual area (values in square centimeter) representing the measured adipose tissue in the entire compartment.

Moreover, the BMI at time of the WBLDCT was gathered in order to investigate its correlation with disease activity, adverse cytogenetics, adverse events and treatment response.

The investigator was blinded with regard to patients´ disease activity, adverse cytogenetics, adverse events and treatment response.

### Disease activity and adverse cytogenetics

Monoclonal protein, bone marrow plasma cells, lactate dehydrogenase (LDH), ISS and CRAB-criteria [20] (calcium, creatinine, hemoglobin and osteopenia/osteolyses/fractures) were chosen to represent the current disease activity. Bone related disease activity for the CRAB-criteria were provided by the radiological reports from the WBLDCTs. In order to detect cytogenetic abnormalities, Interphase fluorescence in situ hybridization analysis was performed on CD138-purified plasma cells. Deletion 17p13, translocation t(4;14) and gain 1q21 >3 copies were defined as adverse cytogenetics. [21] Deletion 13q14 was additionally evaluated in this study.

### Adverse events and treatment response

Adverse events were graded using the CTCAE catalogue, version 4.0, of the National Institutes of Health (NIH). [22] Adverse events analyzed in this study were infections (CTC grade 2 or greater), leukopenia (CTC grade 3 or greater), thrombocytopenia (CTC grade 3 or greater) and thromboembolism (CTC grade 2 or greater). Treatment response was assessed according to the International Myeloma Working Group (IMWG) Uniform Response Criteria, modified to include near complete response. [23] Patients achieving at least a very good partial response (VGPR) after induction therapy were classified as responders.

### Statistical analysis

In a first step, univariate analyses were used to get a first impression of the effects of the fat parameters on treatment response and to identify possible confounders. The parameters were divided into continuous variables (monoclonal protein, LDH, plasma cells, calcium, creatinine, hemoglobin, BMI) and categorical variables (ISS, osteopenia, osteolysis, fracture, del 17p13, t(4;14), gain 1q21, del 13q14, treatment response, infection, leukopenia, thrombocytopenia, thromboembolism) for the statistical analysis. The continuous variables were analysed using the Pearson correlation or the Spearman correlation respectively in case of non-normally distributed variables. The single factor analysis of variance (univariate ANOVA or Kruskal-Wallis test) was used for categorical variables. *P* values lower than 0.05 were considered statistically significant. Due to the explorative character of these investigations, correction for multiple testing was omitted.

To further investigate the association between the body fat composition and the treatment response in the presence of confounders and to be able to account for the correlation structure between the fat parameters, a multivariate logistic regression model was fitted. Revised ISS (combination of ISS, cytogenetics and LDH) [13] and treatment arm (VCD/PAD) were included into the model as relevant prognostic factors. Fat parameters (VAT and SAT of the abdomen and pelvis and TAT of the thigh compartment) with an additional effect on the response to treatment even in the presence of confounders were determined using backward selection. That is, the fat parameters were excluded from the full model in a stepwise fashion until the p-values for all remaining fat parameters in the model are less than 0.1. Instead of the usual significance level of 0.05, the cut-off of 0.1 was chosen to overcome the lack of power to detect a true predictor due to small sample size.

For validation of the variable selection method, the stability of the selection algorithm was investigated by means of bootstrapping. Therefore, 500 bootstrap samples of 94 patients each (number of complete observations for the multivariate model) were drawn with replacement from the original data set and for each sample, the variable selection method was conducted. Selection frequencies for each of the fat parameters were estimated by the proportion of bootstrap replicates in which the corresponding variable was selected.

All analyses were performed with software R, Version 3.0.2 (R Foundation, Vienna, Austria) and SPSS (IBM, Armonk, USA). The multivariate analysis was performed with R, Version 3.3.1.

## Abbreviations

BAFF: B-cell Acitivating Factor
BMI: body mass index
GMMG: German-Speaking Myeloma Multicenter Group
HU: Hounsfield Units
IMWG: International Myeloma Working Group
ISS: International Staging System
LDH: lactate dehydrogenase
MM: multiple myeloma
PAd: bortezomib, adriamycin, dexamethasone
ROI: region of interest
SAT: subcutaneous adipose tissue
TAT: total adipose tissue
VAT: visceral adipose tissue
VCD: bortezomib, cyclophosphamide, dexame-thasone
VGPR: very good partial response
WBLDCT: whole-body low-dose computed tomography
